# Exploration and machine learning model development for T2 NSCLC with bronchus infiltration and obstructive pneumonia/atelectasis

**DOI:** 10.1038/s41598-024-55507-6

**Published:** 2024-02-27

**Authors:** Xuanhong Jin, Yang Pan, Chongya Zhai, Hangchen shen, Liangkun You, Hongming Pan

**Affiliations:** 1 Department of Medical Oncology, Sir Run Run Shaw Hospital, College of Medicine, Zhejiang University, Hangzhou, China; 2 Postgraduate Training Base Alliance of Wenzhou Medical University (Zhejiang Cancer Hospital), Hangzhou, China; 3 Hangzhou Institute of Medicine (HIM), Chinese Academy of Sciences, Hangzhou, China

**Keywords:** Cancer models, Lung cancer

## Abstract

In the 8th edition of the American Joint Committee on Cancer (AJCC) staging system for Non-Small Cell Lung Cancer (NSCLC), tumors exhibiting main bronchial infiltration (MBI) near the carina and those presenting with complete lung obstructive pneumonia/atelectasis (P/ATL) have been reclassified from T3 to T2. Our investigation into the Surveillance, Epidemiology, and End Results (SEER) database, spanning from 2007 to 2015 and adjusted via Propensity Score Matching (PSM) for additional variables, disclosed a notably inferior overall survival (OS) for patients afflicted with these conditions. Specifically, individuals with P/ATL experienced a median OS of 12 months compared to 15 months (p < 0.001). In contrast, MBI patients demonstrated a slightly worse prognosis with a median OS of 22 months versus 23 months (p = 0.037), with both conditions significantly correlated with lymph node metastasis (All p < 0.001). Upon evaluating different treatment approaches for these particular T2 NSCLC variants, while adjusting for other factors, surgery emerged as the optimal therapeutic strategy. We counted those who underwent surgery and found that compared to surgery alone, the MBI/(P/ATL) group experienced a much higher proportion of preoperative induction therapy or postoperative adjuvant therapy than the non-MBI/(P/ATL) group (41.3%/54.7% vs. 36.6%). However, for MBI patients, initial surgery followed by adjuvant treatment or induction therapy succeeded in significantly enhancing prognosis, a benefit that was not replicated for P/ATL patients. Leveraging the XGBoost model for a 5-year survival forecast and treatment determination for P/ATL and MBI patients yielded Area Under the Curve (AUC) scores of 0.853 for P/ATL and 0.814 for MBI, affirming the model's efficacy in prognostication and treatment allocation for these distinct T2 NSCLC categories.

## Introduction

Non-small cell lung cancer (NSCLC) stands as one of the predominant contributors to global cancer-related mortality rates^1–3^. Up to 70% of NSCLC patients are diagnosed at advanced stages, of which the median overall 5-year overall survival (OS) rate is substantially low, at 4% to 6%^4,5^.

In the 8th edition of the AJCC staging system, the criteria for T2 stage tumors have been refined, reducing the size threshold from 7 to 5 cm. Presently, tumors exhibiting local extension are classified as T2. Local extension denotes scenarios such as obstructive pneumonitis/atelectasis (P/ATL) impacting either a segment or the entire lung. This category also encompasses neoplasms that penetrate the visceral pleura and those affecting the main bronchus, irrespective of their proximity to the carina, provided the carina is not directly implicated^6^. This change reflects the prognostic value of main bronchus infiltration (MBI) and P/ATL^7–9^.

In patients with advanced lung cancer, common metastatic sites include the lung, lymph nodes, brain, bones, adrenal glands, and liver. The cancer often spreads to multiple areas, with survival varying due to biological differences and treatment approaches^10,11^. Notably, in clinical practice, lymph node metastasis is regarded as one of the most important prognostic factors for NSCLC patients^12,13^. A study found that P/ATL is a risk factor for lymph node metastasis^14^. However, the relationship between MBI and P/ATL with lymph node metastasis still needs further exploration.

Furthermore, these two distinct subtypes of stage T2 NSCLC may pose more significant treatment challenges compared to standard T2 tumors. Despite this, there has been a lack of thorough research to determine the optimal treatment approach for these specific T2 NSCLC subtypes. The effectiveness and role of treatment modalities, in the context of the two specific NSCLC subtypes, remain to be clarified.

Employing machine learning, a branch of artificial intelligence, for model development enhances the accuracy of predictions with the addition of new data, frequently outperforming logistic regression methods. Its application in predicting survival across various cancers has been noted, and specifically, utilizing machine learning to estimate 5-year OS for T2-stage NSCLC patients under certain conditions significantly improves the precision of prognosis forecasts^15,16^. This approach not only refines survival predictions but also facilitates the formulation of recommendations for optimal treatment strategies.

## Results

### Clinical characteristics of T2 stage NSCLC patients in different groups

Variations in clinical characteristics between the MBI/(P/ATL) and non-MBI/(P/ATL) groups were prominently attributed to the diameter linked to the T2 stage **(**Table 1**)**. Notable disparities existed in gender distribution, with the MBI/(P/ATL) group demonstrating a higher proportion of males (58.4%/55.3% vs. 53.4%) and a heightened occurrence of Squamous Cell Carcinoma (46.0%/40.8% vs. 32.7%). Significantly, a larger proportion of primary sites in the main bronchus were identified in the MBI/(P/ATL) group (14.1%/7.8% vs. 1.7%), accompanied by a more advanced histologic grading (p < 0.001).

**Table 1:** Patient characteristics.

	None	MBI	P/ATL	P-value
	(N = 20,543)	(N = 2718)	(N = 3572)	
Age				
≥ 75	7539 (36.7%)	710 (26.1%)	1098 (30.7%)	< 0.001
65–74	7167 (34.9%)	927 (34.1%)	1205 (33.7%)	
30–64	5837 (28.4%)	1081 (39.8%)	1269 (35.5%)	
Sex				
Male	10,972 (53.4%)	1588 (58.4%)	1974 (55.3%)	< 0.001
Female	9571 (46.6%)	1130 (41.6%)	1598 (44.7%)	
Race				
White	17,068 (83.1%)	2301 (84.7%)	2933 (82.1%)	< 0.001
Black	2015 (9.8%)	311 (11.4%)	441 (12.3%)	
Asian	1460 (7.1%)	106 (3.9%)	198 (5.5%)	
Histologic type				
AD	10,715 (52.2%)	1004 (36.9%)	1519 (42.5%)	< 0.001
SQCC	6724 (32.7%)	1249 (46.0%)	1458 (40.8%)	
LCC	1868 (9.1%)	285 (10.5%)	386 (10.8%)	
Others	1236 (6.0%)	180 (6.6%)	209 (5.9%)	
Grade				
I	2040 (9.9%)	199 (7.3%)	248 (6.9%)	< 0.001
II	7431 (36.2%)	995 (36.6%)	1212 (33.9%)	
III	10,553 (51.4%)	1455 (53.5%)	2006 (56.2%)	
IV	519 (2.5%)	69 (2.5%)	106 (3.0%)	
N				
N0	10,896 (53.0%)	1135 (41.8%)	1215 (34.0%)	< 0.001
N1	2512 (12.2%)	472 (17.4%)	445 (12.5%)	
N2	5618 (27.3%)	884 (32.5%)	1506 (42.2%)	
N3	1517 (7.4%)	227 (8.4%)	406 (11.4%)	
M				
M0	14,488 (70.5%)	1967 (72.4%)	2002 (56.0%)	< 0.001
M1	6055 (29.5%)	751 (27.6%)	1570 (44.0%)	
Site				
Upper lobe	12,019 (58.5%)	1441 (53.0%)	1945 (54.5%)	< 0.001
Lower lobe	7058 (34.4%)	723 (26.6%)	1088 (30.5%)	
Middle lobe	931 (4.5%)	131 (4.8%)	215 (6.0%)	
Main bronchus	344 (1.7%)	382 (14.1%)	280 (7.8%)	
Overlapping lesion	191 (0.9%)	41 (1.5%)	44 (1.2%)	
Laterality				
Right	11,946 (58.2%)	1615 (59.4%)	2037 (57.0%)	0.486
Left	8597 (41.8%)	1103 (40.6%)	1535 (43.0%)	
Size				
Mean (SD)	39.8 (5.81)	34.5 (10.5)	34.3 (11.0)	< 0.001
Median [min, max]	40.0 [31.0, 50.0]	35.0 [2.00, 50.0]	35.0 [1.00, 50.0]	
Radiation				
No/unknown	12,691 (61.8%)	1543 (56.8%)	1901 (53.2%)	< 0.001
Yes	7852 (38.2%)	1175 (43.2%)	1671 (46.8%)	
Chemotherapy				
No/unknown	11,978 (58.3%)	1414 (52.0%)	1745 (48.9%)	< 0.001
Yes	8565 (41.7%)	1304 (48.0%)	1827 (51.1%)	
Surgery				
Surgery alone	6006 (29.2%)	795 (29.2%)	429 (12.0%)	< 0.001
Induction therapy followed by surgery	426 (2.1%)	76 (2.8%)	72 (2.0%)	
Initial surgery followed by adjuvant treatment	3038 (14.8%)	484 (17.8%)	446 (12.5%)	
None	11,073 (53.9%)	1363 (50.1%)	2625 (73.5%)	
Marital status				
Married	10,939 (53.2%)	1496 (55.0%)	1860 (52.1%)	0.193
Unmarried/others	9604 (46.8%)	1222 (45.0%)	1712 (47.9%)	

MBI: Main Bronchus Infiltration, P/ATL: Obstructive Pneumonia/Atelectasis, SD: Standard Deviation, AD: Adenocarcinoma, SQCC: Squamous Cell Carcinoma, LCC: Large Cell Carcinoma.

The MBI/(P/ATL) group, especially the P/ATL subgroup, exhibited higher incidences of lymph nodes (N0: 41.8%/34.0% vs. 53.0%). Regarding treatment modalities, the MBI/(P/ATL) group displayed a stronger propensity to undergo chemotherapy (48.0%/51.1% vs. 41.7%) and radiation therapy (43.2%/46.8% vs. 38.2%). Compared to MBI/None group, the incidence of surgery was markedly lower in the P/ATL subgroup (26.5% vs. 49.9%/46.1%). Moreover, we counted those who underwent surgery and found that compared to surgery alone, the MBI/(P/ATL) group experienced a much higher proportion of preoperative induction therapy or postoperative adjuvant therapy than the non-MBI/(P/ATL) group (41.3%/54.7% vs. 36.6%).

In relation to tumor diameter, the non-MBI/(P/ATL) group had a larger diameter due to the incorporation of cases surpassing 3 cm. In general, profound differences in clinical characteristics were observed between the groups, with the MBI/(P/ATL) group manifesting extensive disparities, especially within the P/ATL subgroup, compared to the non-MBI/(P/ATL) group.

### Survival analysis before and after PSM

**Figure 1:**
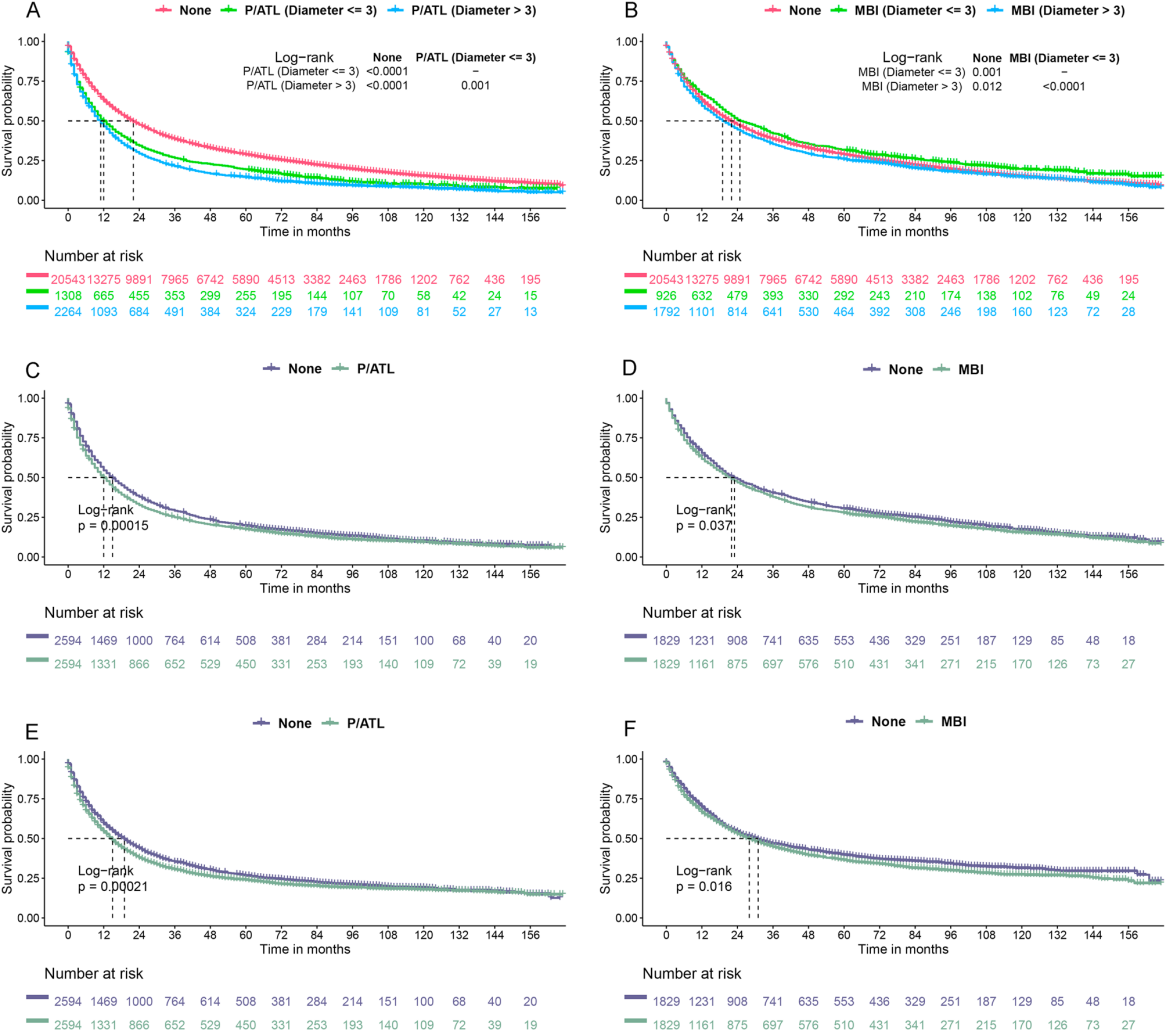
Kaplan–Meier analysis of patients with different T2 types of NSCLC. (**A**,**B**) Kaplan–Meier analysis of overall survival (OS) in the Pneumonia or Atelectasis (P/ATL) and Main Bronchus Infiltration (MBI) groups versus the groups without P/ATL and MBI, prior to propensity score matching (PSM). (**C**,**D**) Kaplan–Meier analysis of OS in the P/ATL and MBI groups versus the non-MBI and P/ATL groups following PSM. (**E**,**F**) Kaplan–Meier analysis of cancer-specific survival (CSS) in the P/ATL and MBI groups versus the non-MBI and P/ATL groups after PSM.

Through Kaplan–Meier survival analysis, it was discerned that the OS for the MBI (Diameter > 3) group was adversely impacted in comparison to the non-MBI/(P/ATL) group (p = 0.012) (Fig. 1A). Notably, regardless of the diameter size, the OS for the non-MBI/(P/ATL) group was significantly superior to that of the P/ATL group (p < 0.0001) (Fig. 1B).

Given the pronounced heterogeneity in clinical characteristics among the three groups, we adopted the Propensity Score Matching (PSM) method to mitigate the impact of diverse background variables, thereby harmonizing potential prognostic factors between the P/ATL and MBI groups compared to the non-MBI/(P/ATL) group. This approach ensured that the p-values from t-tests or chi-square tests for all clinical characteristics between the respective groups exceeded 0.1, indicating a balanced comparison (Supplementary data 1). Following this adjustment, we analyzed OS and cancer-specific survival (CSS) using the KM method for the P/ATL vs. None groups and the MBI vs. None groups, respectively. Our findings revealed that the P/ATL group exhibited a significantly poorer prognosis than the None group, with p of 0.00015 for OS and 0.00021 for CSS (Fig. 1C,E). Conversely, the MBI group's prognosis was marginally inferior compared to the None group, with p of 0.037 for OS and 0.016 for CSS (Fig. 1D,F).

### Multivariate logistic regression analysis for lymph node metastasis

Our findings indicate that at the T2 stage, both the MBI and P/ATL groups demonstrate an elevated risk for lymph node metastasis. To ascertain whether MBI and P/ATL act as independent risk factors for these lymph node metastase, we employed a multifactorial logistic regression analysis. The results illuminated those individuals in the MBI/(P/ATL) group had a notably higher risk of lymph node metastasis compared to those in the non-MBI/(P/ATL) group. In detail, MBI was found to be an independent risk factor for lymph node metastasis (OR = 1.69, 95% CI 1.55–1.85, p < 0.001), as was P/ATL (OR = 2.10, 95% CI 1.93–2.28, p < 0.001) **(**Table 2**)**.

**Table 2:** Multivariate logistic regression analysis of potential predictors for lymph node metastasis in T2 NSCLC.

Characteristics	Multivariate analysis	
	HR (95%CI)	P‐value
Age		
≥ 75	Reference	
65–74	1.22 (1.14–1.29)	P < 0.001
30–64	1.48 (1.38–1.57)	P < 0.001
Sex		
Male	Reference	
Female	0.98 (0.92–1.03)	P = 0.364
Race		
White	Reference	
Black	1.17 (1.07–1.28)	P < 0.001
Asian	1.10 (0.99–1.22)	P = 0.071
Histologic type		
AD	Reference	
SQCC	0.90 (0.85–0.95)	P < 0.001
LCC	1.07 (0.98–1.18)	P = 0.147
Others	0.96 (0.86–1.07)	P = 0.441
Grade		
I	Reference	
II	2.03 (1.83–2.24)	P < 0.001
III	2.88 (2.60–3.18)	P < 0.001
IV	2.50 (2.07–3.02)	P < 0.001
M		
M0	Reference	
M1	3.03 (2.86–3.21)	P < 0.001
Site		
Upper lobe	Reference	
Lower lobe	0.97 (0.91–1.02)	P = 0.227
Middle lobe	0.93 (0.82–1.05)	P = 0.250
Main bronchus	1.48 (1.28–1.72)	P < 0.001
Overlapping lesion	1.00 (0.77–1.28)	P = 0.972
Laterality		
Right	Reference	
Left	0.90 (0.85–0.95)	P < 0.001
Size		
Mean ± SD	1.02 (1.02–1.02)	P < 0.001
Marital status		
Married	Reference	
Unmarried/others	0.92 (0.88–0.97)	P = 0.003
Type		
None	Reference	
P/ATL	2.10 (1.93–2.28)	P < 0.001
MBI	1.69 (1.55–1.85)	P < 0.001

MBI: main bronchus infiltration, P/ATL: obstructive pneumonia/atelectasis, SD: Standard deviation, AD: adenocarcinoma, SQCC: squamous cell carcinoma, LCC: large cell carcinoma.

### Evaluation of different treatments in patients with MBI and P/ATL

To evaluate the optimal treatment for NSCLC patients with two specific types of T2 tumors, we integrated seven treatment modalities: None, Radiation Therapy Alone, Chemotherapy Alone, Radiation + Chemotherapy, Surgery Alone, Initial Surgery Followed by Adjuvant Treatment, and Induction Therapy Followed by Surgery. We conducted a multifactorial Cox regression analysis of OS to assess the prognostic impact of these treatments in patients with P/ATL and MBI, respectively, using Surgery Alone as the reference group **(**Table 3**)**. The results indicated that surgical treatments significantly outperformed both Radiotherapy Alone and Chemotherapy Alone, as well as the combination of Radiotherapy and Chemotherapy, in both subgroups. Specifically, in patients with MBI, Initial Surgery Followed by Adjuvant Treatment (HR = 0.77, 95% CI 0.67–0.90, p = 0.001) and Induction Therapy Followed by Surgery (HR = 0.65, 95% CI 0.48–0.87, p = 0.003) were significantly more effective than Surgery Alone. Conversely, for patients with P/ATL, neither Initial Surgery Followed by Adjuvant Treatment (HR = 1.17, 95% CI 0.99–1.37, p = 0.067) nor Induction Therapy Followed by Surgery (HR = 1.05, 95% CI 0.78–1.40, p = 0.758) showed any advantage over Surgery Alone.

**Table 3:** Multivariate cox regression analysis of OS within MBI group and P/ATL group.

Characteristics	MBI patients		P/ATL patients	
	HR (95%CI)	P‐value	HR (95%CI)	P‐value
Age				
≥ 75	Reference		Reference	
65–74	0.79 (0.71–0.88)	P < 0.001	0.89 (0.82–0.97)	P = 0.011
30–64	0.67 (0.61–0.75)	P < 0.001	0.81 (0.74–0.88)	P < 0.001
Sex				
Male	Reference		Reference	
Female	0.76 (0.69–0.83)	P < 0.001	0.88 (0.82–0.95)	P = 0.001
Race				
White	Reference		Reference	
Black	1.03 (0.90–1.17)	P = 0.705	0.95 (0.85–1.05)	P = 0.315
Asian	0.69 (0.55–0.88)	P = 0.002	0.71 (0.60–0.83)	P < 0.001
Histologic type				
AD	Reference		Reference	
SQCC	0.99 (0.90–1.09)	P = 0.830	0.97 (0.89–1.05)	P = 0.468
LCC	1.04 (0.90–1.21)	P = 0.580	1.36 (1.20–1.53)	P < 0.001
Others	0.73 (0.60–0.89)	P = 0.002	1.02 (0.87–1.20)	P = 0.810
Grade				
I	Reference		Reference	
II	1.27 (1.06–1.53)	P = 0.012	1.19 (1.02–1.39)	P = 0.027
III	1.40 (1.16–1.68)	P < 0.001	1.31 (1.13–1.52)	P < 0.001
IV	1.77 (1.29–2.44)	P < 0.001	1.37 (1.07–1.76)	P = 0.012
N				
N0	Reference		Reference	
N1	1.45 (1.27–1.65)	P < 0.001	1.19 (1.06–1.35)	P = 0.005
N2	1.82 (1.63–2.04)	P < 0.001	1.46 (1.33–1.59)	P < 0.001
N3	1.77 (1.50–2.10)	P < 0.001	1.58 (1.39–1.79)	P < 0.001
M				
M0	Reference		Reference	
M1	1.94 (1.75–2.16)	P < 0.001	1.82 (1.68–1.97)	P < 0.001
Site				
Upper lobe	Reference		Reference	
Lower lobe	1.11 (1.00–1.22)	P = 0.048	0.93 (0.86–1.01)	P = 0.091
Middle lobe	1.07 (0.87–1.31)	P = 0.534	0.99 (0.85–1.15)	P = 0.875
Main bronchus	1.10 (0.97–1.24)	P = 0.144	1.08 (0.95–1.24)	P = 0.229
Overlapping lesion	0.90 (0.63–1.28)	P = 0.548	1.03 (0.74–1.42)	P = 0.872
Laterality				
Right	Reference		Reference	
Left	1.00 (0.92–1.09)	P = 0.977	0.99 (0.92–1.06)	P = 0.795
Size				
Mean ± SD	1.01 (1.00–1.01)	P = 0.002	1.01 (1.00–1.01)	P = 0.001
TreatmentType				
Surgery alone	Reference		Reference	
None	4.38 (3.72–5.16)	P < 0.001	5.02 (4.32–5.84)	P < 0.001
Radiation therapy alone	2.63 (2.26–3.06)	P < 0.001	3.41 (2.93–3.98)	P < 0.001
Chemotherapy alone	1.50 (1.24–1.82)	P < 0.001	2.01 (1.71–2.38)	P < 0.001
Radiation + chemotherapy	1.60 (1.39–1.84)	P < 0.001	1.74 (1.50–2.03)	P < 0.001
Initial surgery followed by adjuvant treatment	0.77 (0.67–0.90)	P = 0.001	1.17 (0.99–1.37)	P = 0.067
Induction therapy followed by surgery	0.65 (0.48–0.87)	P = 0.003	1.05 (0.78–1.40)	P = 0.758
Marital status				
Married	Reference		Reference	
Unmarried/others	1.19 (1.09–1.30)	P < 0.001	1.11 (1.04–1.20)	P = 0.004

MBI: main bronchus infiltration, P/ATL: obstructive pneumonia/atelectasis, SD: standard deviation, AD: adenocarcinoma, SQCC: squamous cell carcinoma, LCC: large cell carcinoma.

**Figure 2:**
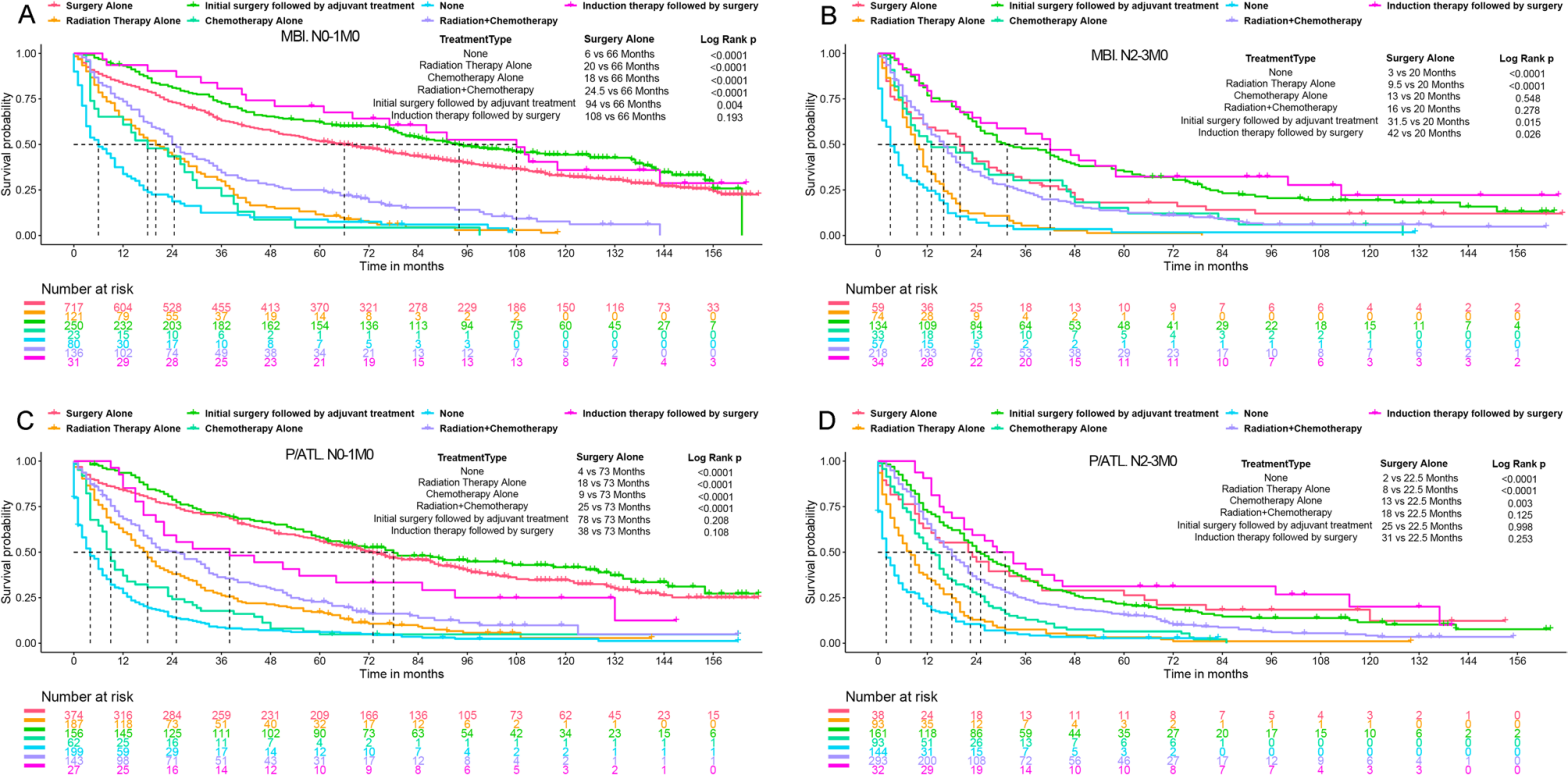
Kaplan–Meier analysis comparing the effectiveness of various treatment modalities in patients with Main Bronchus Infiltration (MBI) or Pneumonia/Atelectasis (P/ATL) based on nodal involvement. (**A**) Overall Survival (OS) associated with different treatment approaches in MBI patients classified as N0-1M0. (**B**) OS associated with different treatment approaches in MBI patients classified as N2-3M0. (**C**) OS associated with different treatment approaches in P/ATL patients classified as N0-1M0. (**D**) OS associated with different treatment approaches in P/ATL patients classified as N2-3M0.

Given the limited therapeutic options for patients with distant metastases, we analyzed the KM survival with different therapeutic strategies for patients with P/ATL and MBI at stages N0-1M0 and N2-3M0, respectively. In patients with MBI at the N2-3M0 stage, preoperative Induction Therapy significantly improved prognosis, illustrating a marked enhancement in outcomes. For the N0-1M0 stage in MBI patients, while there was a clear improvement in median survival with preoperative Induction Therapy, this improvement did not reach statistical significance. Additionally, postoperative Adjuvant Therapy substantially improved outcomes over Surgery Alone for MBI patients across both N0-1M0 and N2-3M0 stages (Fig. 2A,B). Conversely, these treatments did not yield significant benefits for patients with P/ATL (Fig. 2C,D). Moreover, in both subgroups for the N0-1M0 stage, prognosis following Surgery Alone was significantly better than with Chemoradiotherapy, whereas at the N2-3M0 stage, Surgery Alone did not show superiority over Chemoradiotherapy in terms of prognosis (Fig. 2).

### Development of predictive models for 5-year OS in P/ATL and MBI patients

Given the potential notable disparities in clinicopathologic variables and prognoses across the MBI and P/ATL subgroups, we aimed to delve deeper into the varying impacts that different factors might exhibit on mortality within these subgroups. Accordingly, multifactorial logistic regression was applied to analyze the 5-year OS rate within the MBI and P/ATL subgroups. In the MBI group, sex, histologic type, grade, age, N stage, M stage, site, marital status and treatment type were identified as independent factors associated with 5-year OS. In the P/ATL group, histologic type, grade, age, race, N stage, M stage and treatment type were recognized as independent factors associated with 5-year OS (Supplementary data 2).

**Figure 3:**
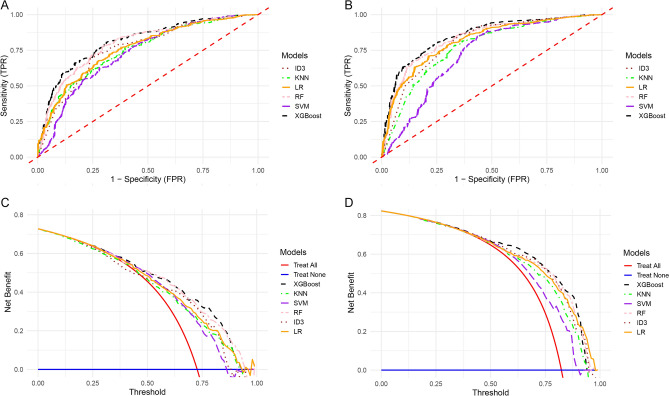
Receiver Operating Characteristic Curve (ROC) and Decision Curve Analysis (DCA) analyses of Main Bronchus Infiltration (MBI) and Pneumonia/Atelectasis (P/ATL) groups. (**A**) ROC curves for each model in the MBI group. (**B**) ROC curves for each model in the P/ATL group. (**C**) DCA curves for each model in the MBI group. (**D**) DCA curves for each model in the P/ATL group.

We incorporated the factors independently correlated with 5-year OS from the MBI and P/ATL groups for prognostic modeling. The patients were randomized into training and test data groups at a 7:3 ratio. Subsequently, the best parameters for each model were adjusted and training was conducted within the training set to optimize performance. In the validation set, we performed ROC and DCA analyses of MBI and P/ATL groups for all models (Fig. 3A,B). The XGBoost model also demonstrated optimal AUC with 0.814 and 0.853 respectively in both MBI and P/ATL groups, and the DCA curves further affirmed that the XGBoost model secures a higher net benefit compared to other models across varying threshold ranges (Fig. 3C,D). The specific performance of each model in the test set is shown in Supplementary Data 3. In addition, we performed the Delong test and found that the XGBoost model significantly outperforms the rest of the models in both MBI and P/ATL (Supplementary Data 4).

**Figure 4:**
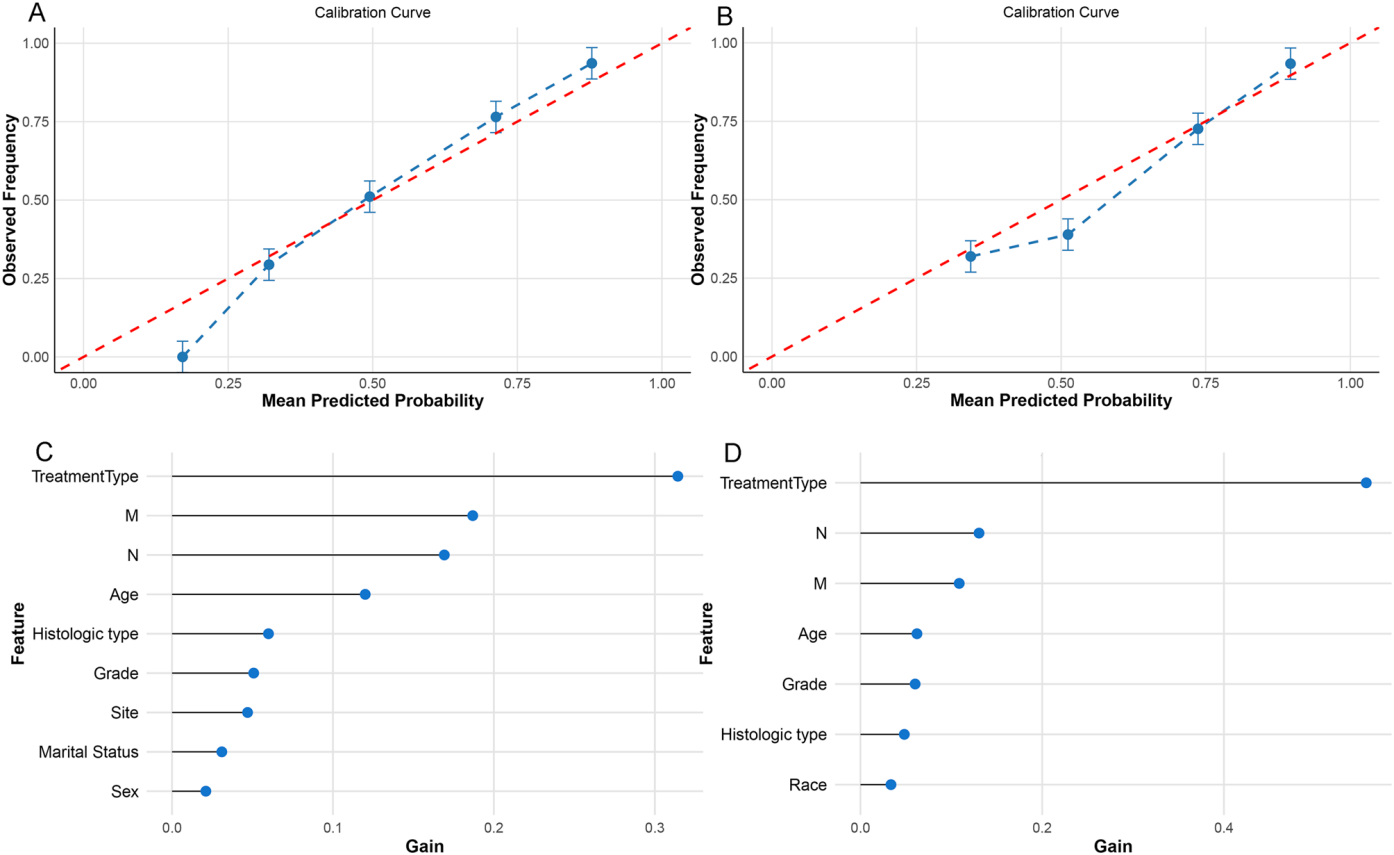
Calibration curves and feature significance plots of the XGBoost model for Main Bronchus Infiltration (MBI) and Pneumonia/Atelectasis (P/ATL) groups. (**A**) Calibration curve of the XGBoost model for the MBI group. (**B**) Calibration curve of the XGBoost model for the P/ATL group. (**C**) Feature significance plot of the XGBoost model for the MBI group. (**D**) Feature significance plot of the XGBoost model for the P/ATL group.

Consequently, the calibration curves for the XGBoost model in both the MBI and P/ATL groups within the test set were also plotted, revealing commendable predictive performance of the model (Fig. 4A,B). Additionally, we scrutinized the importance scores of the variables in both models (Fig. 4C,D).

### Creating web-based predictive models

**Figure 5:**
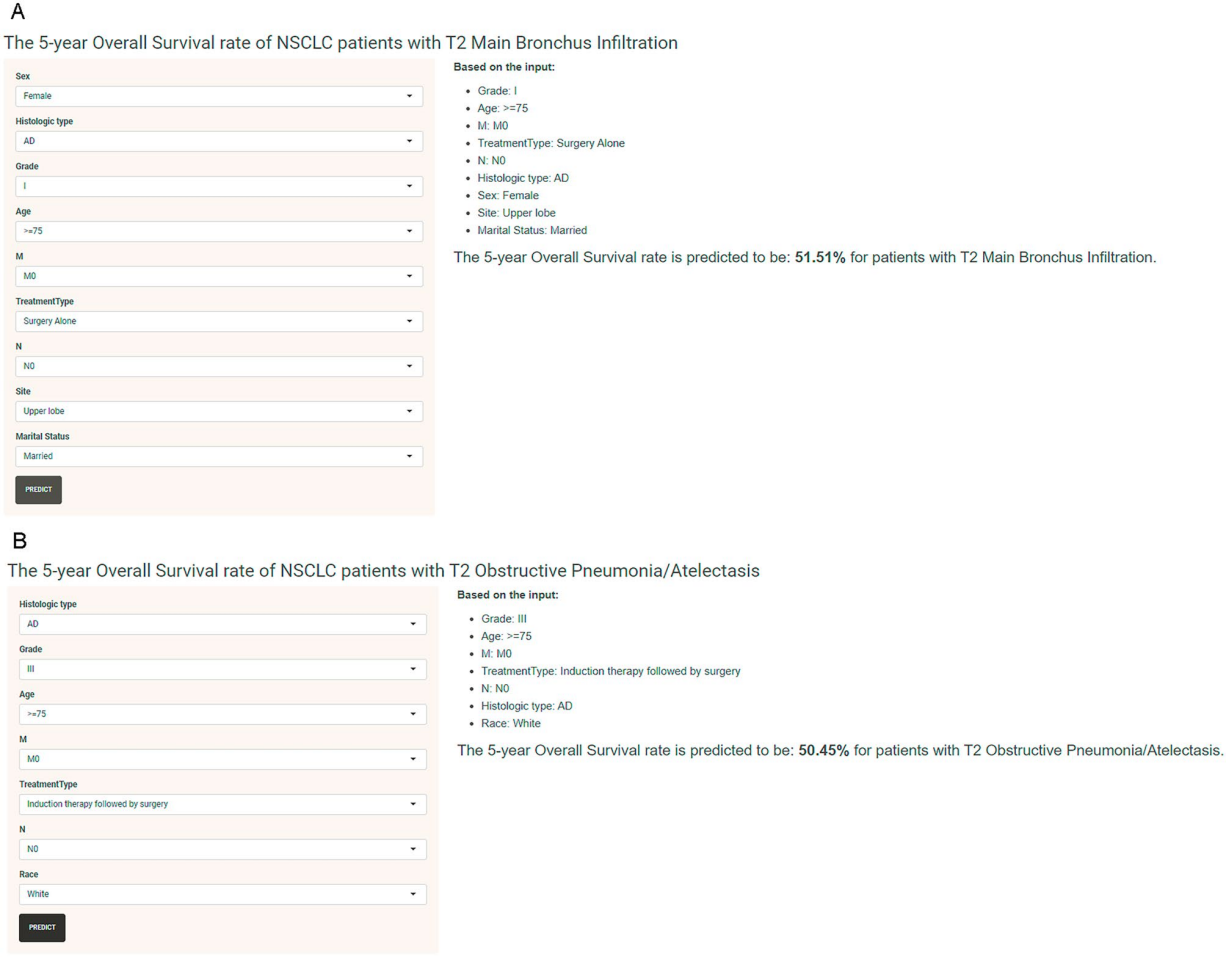
Web applications for T2 NSCLC MBI and P/ATL groups. (**A**) https://medicalresearchapp.shinyapps.io/MBI_5_years_death/. (**B**) https://medicalresearchapp.shinyapps.io/P_ATL_5_years_death/.

To assist researchers and clinicians in utilizing our prognostic model, we developed user-friendly web applications for stage T2 NSCLC MBI and P/ATL groups (Fig. 5A,B), respectively. The web interface allows users to input clinical features of new samples, and the application can then help predict survival probabilities and survival status based on the patient's information. And the model can help clinicians to develop appropriate treatment strategies for this subgroup of patients by first selecting other parameters of a particular patient and focusing on the change of their 5-year survival by adjusting different treatments. For example, a 65–74 year old male with T2N3M0 stage lung adenocarcinoma, graded as grade III located in the upper lobe of a married MBI patient, his 5-year OS was 19.07% if he received Chemoradiotherapy, 23.83% if he received only surgery, and 5-year OS if he received Induction therapy followed by surgery was 35.51%, and 31.28% for those who received Initial surgery followed by adjuvant treatment.

## Discussion

Although there are many studies examining NSCLC, studies specifically examining specific types of T2-staged NSCLC are still very limited currently. We performed the first comprehensive analysis of T2 stage NSCLC in MBI as well as P/ATL subgroups. Previous research, relying solely on the Cox proportional hazards model, has indicated that P/ATL may have an independent prognostic impact on stage T2 NSCLC^9,17^. However, when considering the inclusion of whole-lung pneumonia or atelectasis in T2 stage analysis, this effect might become more pronounced. After adjusting for all other factors through PSM, we observed that patients with P/ATL and MBI, having similar tumor diameters, faced a significantly worse prognosis in T2 stage lung cancer compared to those without these specific conditions. This adverse impact was especially marked in patients with P/ATL. Leveraging the largest sample size to date, our study is the first to confirm the independent effect of P/ATL on prognosis using a PSM approach. In addition, through multivariate logistic regression, we found a significant increase in lymph node metastasis in the P/ATL subgroup compared to the other T2 groups, and found more lymph node metastasis in the MBI subgroup as well, which may be clinically helpful in predicting lymph node metastasis in NSCLC patients.

In addition to this, we compared the treatment options in patients with MBI and P/ATL. We found that surgery remains the treatment of choice for patients with MBI and P/ATL, and that, in patients with MBI, the prognostic impact of preoperative induction therapy and postoperative adjuvant therapy is significant. In P/ATL patients, the proportion of surgical patients was significantly lower, and the proportion of patients receiving simultaneous preoperative induction chemotherapy and postoperative adjuvant therapy was significantly higher than in MBI patients, but the effects of preoperative induction therapy and postoperative adjuvant therapy were poorer in P/ATL patients, and no significant prognostic improvement was found to exist. Earlier research suggested that the P/ATL group might derive greater benefits from radiotherapy^18^, Our study did find that radiotherapy alone had a significantly better prognosis than chemotherapy alone in P/ATL patients with T2N0-1M0 (18 months vs. 9 months), but the role of radiotherapy in higher staged P/ATL patient populations needs to be further elucidated. Moreover, due to the limitations of the SEER database, the impact of further therapies such as targeted and immunotherapies on P/ATL, a group of patients with poorer prognosis, deserves to be further investigated.

In order to accurately predict the prognosis and treatment options for these two subgroups of patients, we embarked on the separate development of machine learning models tailored to each subtype. XGBoost has consistently demonstrated superior predictive performance in various studies^19–21^, and it remained the top performer in our modeling as well. The outcomes indicated that our models achieved superior AUC values relative to preceding prognostic models for NSCLC^22^. This underscores the enhanced predictive accuracy our models offer, particularly for these specialized T2 stage NSCLC categories.

Several limitations merit attention when interpreting the results of this model. Firstly, our study had certain limitations in its scope of variables analyzed, mainly due to the constraints of data availability in the SEER database. As a result, some tumor markers and hematological indicators were omitted. Secondly, detailed information pertaining to the treatment regimen, including specifics on immunotherapy and targeted therapies, was absent. Lastly, it's crucial to note that our model was conceived, ratified, and examined utilizing retrospective data. It's essential that prospective validation studies be conducted to validate our findings before considering its routine application in clinical settings.

## Methods

### Information source and study framework

**Figure 6:**
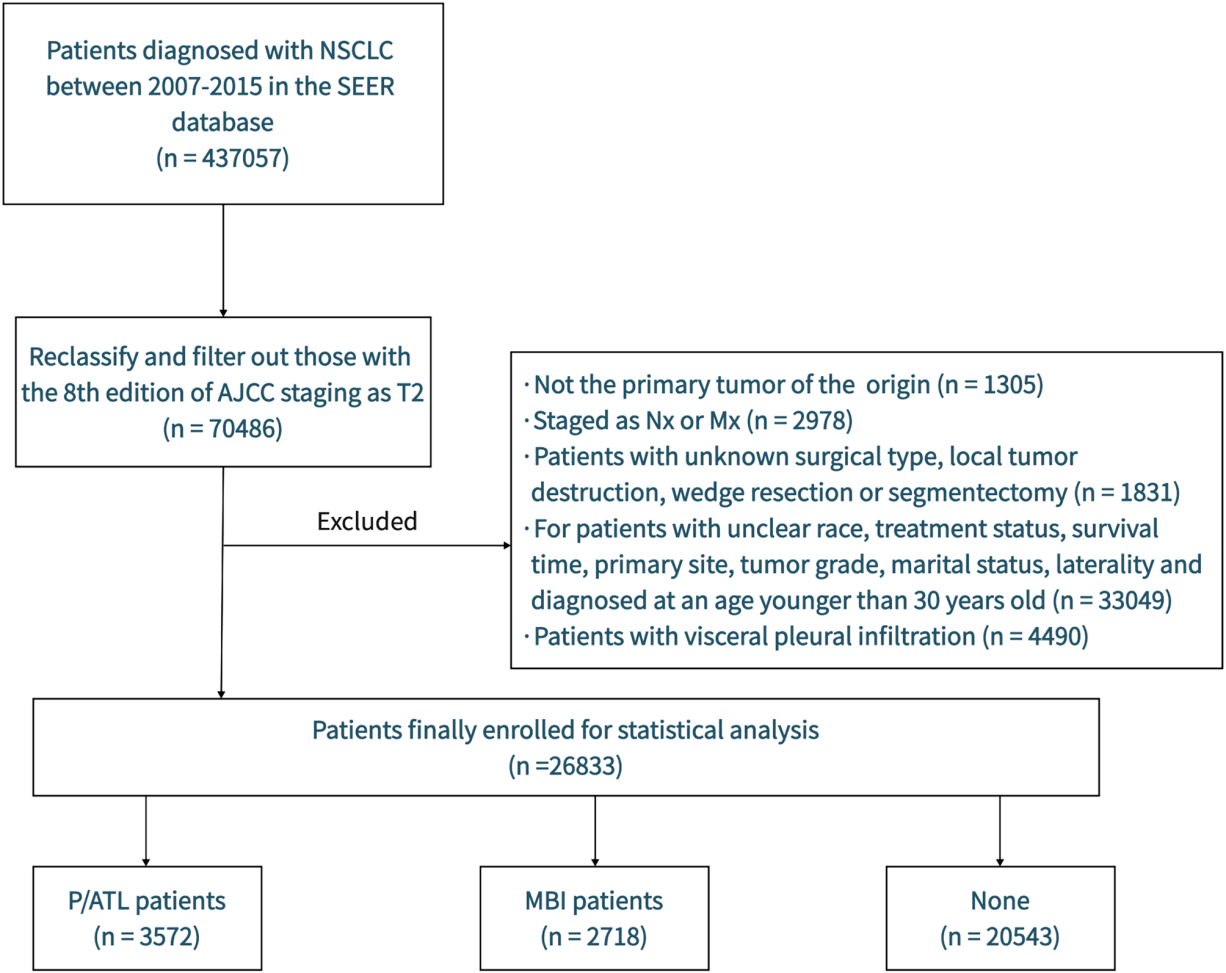
Flow chart of patients’ selection.

The data, representing approximately 27% of the U.S. population, utilized for analysis in this study were sourced from the SEER database [SEER 17 Regs Research Data, Nov 2022 Sub (2000–2020)], a platform where the data are publicly available. We gathered data pertaining to T2 stage NSCLC from 2007 to 2015 from this resource. The criteria for inclusion were as follows: (1) T2 stage NSCLC restaged in accordance with the 8th edition of the AJCC staging; (2) Histological types are restricted to adenocarcinoma (AD) (aligned with SEER histologic codes 8140, 8144, 8230, 8250, 8255, 8260, 8290, 8310, 8323, 8333, 8401, 8480, 8490, 8550, 8570, 8571, 8574), squamous cell carcinoma (SQCC) (specified by histology codes 8052, 8070–8075, 8083, 8084, 8123), large cell carcinoma (LCC) (identified by histology codes 8012–8014, 8031–8033, 8046,8082) and additional varieties of NSCLCs (8022, 8200, 8240, 8430, 8560, 8562, 8980); (3) The lung being the primary site as established by international norms. The exclusion criteria were as follows: (1) Patients demonstrating visceral pleural infiltration; (2) Patients with undefined clinical features. Figure 6 delineates the flowchart of the study..

### Variable selection

Given that the M and N classifications in the SEER database are established at the time of initial diagnosis, our exploration of the association between P/ATL and MBI in lymph node metastasis required a focus on clinical and pathological variables only, such as Size, Marital Status, Primary Site, Sex, Histologic type, Race, Grade, Laterality, and Age, omitting therapeutic variables. However, during the modeling process, all clinical, pathological, and the therapeutic variables were included. In this study, the model is constructed using 5-year OS specifically attributed to cancer. We also collected two ending variables, cancer-specific survival (CSS) and OS. In this study, the OS is based on a 5-year post-diagnosis timeframe. If a patient dies within these 5 years, their OS indicates 'mortality'. However, if a patient survives beyond the 5 years, or has a survival time less than 5 years solely due to the follow-up period, their OS is considered as 'survival'.

### Machine learning model formulation

We utilized multifactorial logistic regression analysis to assess variables and identify independent predictors associated with 5-year OS in MBI or P/ATL in NSCLC. The dataset was randomly split into a 70% training group and a 30% testing group in both MBI and P/ATL groups. Five renowned machine learning models—random forest (RF), K-Nearest Neighbor (KNN), XGBoost, logistic regression (LR), decision tree (ID3), and support vector machine (SVM)—were employed to predict which patients with MBI or P/ATL in NSCLC T2 stage would incur 5-year OS.

During training, we applied lattice filtering and conducted five internal cross-validations to adjust the models' remaining parameters (Supplementary data 5) and performed five external cross-validations to bolster the models’ stability. Models were thoroughly evaluated based on their AUC (Area Under the Curve), specificity, sensitivities, accuracies, correctness and recall in the test set. We compared the performance differences among different models and selected the one with the highest comprehensive score as the final model.

Moreover, utilizing the “shiny” package, we developed two specialized web-based applications to forecast the 5-year OS in patients diagnosed with P/ATL and MBI, respectively.

### Statistical analysis

All data analyses, data visualization, and statistical analyses in this manuscript were performed in R Studio (version 4.2.1). Between-group differences in the P/ATL, MBI, and None groups were tested using the ANOVA test or the chi-square test, with the Bonferroni correction applied for multiple comparisons. Survival analyses for OS were performed using Kaplan–Meier plots and the log-rank test. In multifactorial logistic regression, a Variance Inflation Factor (VIF) of 5 or below was considered indicative of the absence of multicollinearity. All machine learning models in this manuscript were constructed using the “mlr3verse” package, and differences in the AUC between the models were assessed using the Delong test. A p of 0.05 or lower was considered statistically significant.

### Supplementary Information

Supplementary Information 1.

Supplementary Information 2.

Supplementary Information 3.

Supplementary Information 4.

Supplementary Information 5.

## Data Availability

All data here are publicly available in the SEER database (https://seer.cancer.gov/).
