# Supplementary material for: Exploration and machine learning model development for T2 NSCLC with bronchus infiltration and obstructive pneumonia/atelectasis
Source: Sci Rep. 2024 Feb 27;14:4793. doi: 10.1038/s41598-024-55507-6 (PMC10899628; doi:10.1038/s41598-024-55507-6)
Supplement: Supplementary file 1 — Supplementary Information 1. [file 41598_2024_55507_MOESM1_ESM.docx]

**Supplementary data 1.** Patient characteristics after PSM.

Patient characteristics after PSM in the P/ATL group.

|  | No | P/ATL | P-value |
| --- | --- | --- | --- |
|  | (N=2594) | (N=2594) |  |
| Age |  |  |  |
| >=75 | 877 (33.8%) | 844 (32.5%) | 0.582 |
| 65-74 | 882 (34.0%) | 888 (34.2%) |  |
| 30-64 | 835 (32.2%) | 862 (33.2%) |  |
| Sex |  |  |  |
| Male | 1420 (54.7%) | 1417 (54.6%) | 0.956 |
| Female | 1174 (45.3%) | 1177 (45.4%) |  |
| Race |  |  |  |
| White | 2104 (81.1%) | 2127 (82.0%) | 0.416 |
| Black | 316 (12.2%) | 316 (12.2%) |  |
| Asian | 174 (6.7%) | 151 (5.8%) |  |
| Histologic.type |  |  |  |
| AD | 1165 (44.9%) | 1158 (44.6%) | 0.193 |
| SQCC | 955 (36.8%) | 1000 (38.6%) |  |
| LCC | 289 (11.1%) | 286 (11.0%) |  |
| Others | 185 (7.1%) | 150 (5.8%) |  |
| Grade |  |  |  |
| I | 175 (6.7%) | 190 (7.3%) | 0.806 |
| II | 871 (33.6%) | 884 (34.1%) |  |
| III | 1481 (57.1%) | 1453 (56.0%) |  |
| IV | 67 (2.6%) | 67 (2.6%) |  |
| N |  |  |  |
| N0 | 973 (37.5%) | 968 (37.3%) | 0.447 |
| N1 | 327 (12.6%) | 335 (12.9%) |  |
| N2 | 1048 (40.4%) | 1013 (39.1%) |  |
| N3 | 246 (9.5%) | 278 (10.7%) |  |
| M |  |  |  |
| M0 | 1571 (60.6%) | 1549 (59.7%) | 0.552 |
| M1 | 1023 (39.4%) | 1045 (40.3%) |  |
| Site |  |  |  |
| Upper lobe | 1449 (55.9%) | 1451 (55.9%) | 0.836 |
| Lower lobe | 852 (32.8%) | 850 (32.8%) |  |
| Middle lobe | 138 (5.3%) | 151 (5.8%) |  |
| Main bronchus | 123 (4.7%) | 109 (4.2%) |  |
| Overlapping lesion | 32 (1.2%) | 33 (1.3%) |  |
| Laterality |  |  |  |
| Right | 1495 (57.6%) | 1487 (57.3%) | 0.844 |
| Left | 1099 (42.4%) | 1107 (42.7%) |  |
| Size |  |  |  |
| Mean (SD) | 38.4 (5.55) | 38.6 (8.40) | 0.444 |
| Median [Min, Max] | 37.0 [31.0, 50.0] | 40.0 [7.00, 50.0] |  |
| Radiation |  |  |  |
| No/unknown | 1388 (53.5%) | 1368 (52.7%) | 0.597 |
| Yes | 1206 (46.5%) | 1226 (47.3%) |  |
| Chemotherapy |  |  |  |
| No/Unknown | 1319 (50.8%) | 1277 (49.2%) | 0.255 |
| Yes | 1275 (49.2%) | 1317 (50.8%) |  |
| Surgery |  |  |  |
| Surgery Alone | 392 (15.1%) | 367 (14.1%) | 0.794 |
| Induction therapy followed by surgery | 57 (2.2%) | 55 (2.1%) |  |
| Initial surgery followed by adjuvant treatment | 344 (13.3%) | 349 (13.5%) |  |
| None | 1801 (69.4%) | 1823 (70.3%) |  |
| Marital.Status |  |  |  |
| Married | 1330 (51.3%) | 1362 (52.5%) | 0.389 |
| Unmarried/Others | 1264 (48.7%) | 1232 (47.5%) |  |

MBI: Main Bronchus Infiltration, P/ATL: Obstructive Pneumonia/Atelectasis, SD: Standard Deviation, AD: Adenocarcinoma, SQCC: Squamous Cell Carcinoma, LCC: Large Cell Carcinoma.

Patient characteristics after PSM in the MBI group.

|  | No | MBI | P-value |
| --- | --- | --- | --- |
|  | (N=1829) | (N=1829) |  |
| Age |  |  |  |
| >=75 | 590 (32.3%) | 536 (29.3%) | 0.145 |
| 65-74 | 622 (34.0%) | 640 (35.0%) |  |
| 30-64 | 617 (33.7%) | 653 (35.7%) |  |
| Sex |  |  |  |
| Female | 788 (43.1%) | 777 (42.5%) | 0.738 |
| Male | 1041 (56.9%) | 1052 (57.5%) |  |
| Race |  |  |  |
| Asian | 96 (5.2%) | 78 (4.3%) | 0.103 |
| Black | 179 (9.8%) | 211 (11.5%) |  |
| White | 1554 (85.0%) | 1540 (84.2%) |  |
| Histologic.type |  |  |  |
| AD | 759 (41.5%) | 771 (42.2%) | 0.812 |
| SQCC | 772 (42.2%) | 757 (41.4%) |  |
| LCC | 192 (10.5%) | 184 (10.1%) |  |
| Others | 106 (5.8%) | 117 (6.4%) |  |
| Grade |  |  |  |
| I | 148 (8.1%) | 133 (7.3%) | 0.534 |
| II | 679 (37.1%) | 654 (35.8%) |  |
| III | 962 (52.6%) | 997 (54.5%) |  |
| IV | 40 (2.2%) | 45 (2.5%) |  |
| N |  |  |  |
| N0 | 880 (48.1%) | 837 (45.8%) | 0.378 |
| N1 | 269 (14.7%) | 277 (15.1%) |  |
| N2 | 552 (30.2%) | 565 (30.9%) |  |
| N3 | 128 (7.0%) | 150 (8.2%) |  |
| M |  |  |  |
| M0 | 1344 (73.5%) | 1313 (71.8%) | 0.266 |
| M1 | 485 (26.5%) | 516 (28.2%) |  |
| Site |  |  |  |
| Lower lobe | 534 (29.2%) | 564 (30.8%) | 0.852 |
| Main bronchus | 72 (3.9%) | 75 (4.1%) |  |
| Middle lobe | 86 (4.7%) | 85 (4.6%) |  |
| Overlapping lesion | 24 (1.3%) | 24 (1.3%) |  |
| Upper lobe | 1113 (60.9%) | 1081 (59.1%) |  |
| Laterality |  |  |  |
| Left | 751 (41.1%) | 727 (39.7%) | 0.438 |
| Right | 1078 (58.9%) | 1102 (60.3%) |  |
| Size |  |  |  |
| Mean (SD) | 38.6 (5.78) | 38.8 (7.52) | 0.2 |
| Median [Min, Max] | 37.0 [31.0, 50.0] | 40.0 [7.00, 50.0] |  |
| Radiation |  |  |  |
| No/unknown | 1120 (61.2%) | 1095 (59.9%) | 0.417 |
| Yes | 709 (38.8%) | 734 (40.1%) |  |
| Chemotherapy |  |  |  |
| No/Unknown | 1037 (56.7%) | 990 (54.1%) | 0.126 |
| Yes | 792 (43.3%) | 839 (45.9%) |  |
| Surgery |  |  |  |
| Surgery Alone | 554 (30.3%) | 551 (30.1%) | 0.902 |
| Induction therapy followed by surgery | 48 (2.6%) | 51 (2.8%) |  |
| Initial surgery followed by adjuvant treatment | 310 (16.9%) | 325 (17.8%) |  |
| None | 917 (50.1%) | 902 (49.3%) |  |
| Marital.Status |  |  |  |
| Married | 1015 (55.5%) | 993 (54.3%) | 0.485 |
| Unmarried/Others | 814 (44.5%) | 836 (45.7%) |  |

MBI: Main Bronchus Infiltration, P/ATL: Obstructive Pneumonia/Atelectasis, SD: Standard Deviation, AD: Adenocarcinoma, SQCC: Squamous Cell Carcinoma, LCC: Large Cell Carcinoma.
