# Supplementary material for: Exploration and machine learning model development for T2 NSCLC with bronchus infiltration and obstructive pneumonia/atelectasis
Source: Sci Rep. 2024 Feb 27;14:4793. doi: 10.1038/s41598-024-55507-6 (PMC10899628; doi:10.1038/s41598-024-55507-6)
Supplement: Supplementary file 2 — Supplementary Information 2. [file 41598_2024_55507_MOESM2_ESM.docx]

**Supplementary data 2.** Multivariate logistic regression analysis of 5-year OS within MBI group and P/ATL group.

|  | MBI patients | | P/ATL patients | |
| --- | --- | --- | --- | --- |
| Characteristics | HR (95%CI) | P‐value | HR (95%CI) | P‐value |
| Age |  |  |  |  |
| >=75 | Reference |  | Reference |  |
| 65-74 | 0.54 (0.40-0.71) | P<0.001 | 0.68 (0.51-0.91) | P=0.008 |
| 30-64 | 0.41 (0.31-0.55) | P<0.001 | 0.47 (0.35-0.62) | P<0.001 |
| Sex |  |  |  |  |
| Male | Reference |  | Reference |  |
| Female | 0.66 (0.53-0.82) | P<0.001 | 0.84 (0.67-1.04) | P=0.111 |
| Race |  |  |  |  |
| White  Black | Reference |  | Reference |  |
|  | 1.16 (0.84-1.62) | P=0.366 | 0.98 (0.70-1.38) | P=0.928 |
| Asian | 0.76 (0.45-1.28) | P=0.304 | 0.50 (0.33-0.76) | P=0.001 |
| Histologic type |  |  |  |  |
| AD | Reference |  | Reference |  |
| SQCC | 0.94 (0.74-1.20) | P=0.629 | 1.15 (0.90-1.46) | P=0.262 |
| LCC | 1.12 (0.75-1.69) | P=0.573 | 2.02 (1.29-3.17) | P=0.002 |
| Others | 0.64 (0.42-0.97) | P=0.034 | 1.19 (0.76-1.87) | P=0.450 |
| Grade |  |  |  |  |
| I | Reference |  | Reference |  |
| II | 1.98 (1.33-2.93) | P<0.001 | 1.88 (1.28-2.76) | P=0.001 |
| III | 2.29 (1.55-3.39) | P<0.001 | 1.76 (1.21-2.57) | P=0.003 |
| IV | 2.41 (1.14-5.12) | P=0.022 | 1.65 (0.79-3.48) | P=0.185 |
| N |  |  |  |  |
| N0 | Reference |  | Reference |  |
| N1 | 1.99 (1.52-2.61) | P<0.001 | 1.45 (1.07-1.97) | P=0.016 |
| N2 | 3.72 (2.79-4.97) | P<0.001 | 2.53 (1.95-3.29) | P<0.001 |
| N3 | 4.06 (2.28-7.22) | P<0.001 | 4.10 (2.49-6.75) | P<0.001 |
| M |  |  |  |  |
| M0 | Reference |  | Reference |  |
| M1 | 3.54 (2.49-5.04) | P<0.001 | 3.06 (2.32-4.03) | P<0.001 |
| Site |  |  |  |  |
| Upper lobe | Reference |  | Reference |  |
| Lower lobe | 1.41 (1.11-1.78) | P=0.005 | 0.89 (0.70-1.12) | P=0.308 |
| Middle lobe | 1.22 (0.75-1.98) | P=0.432 | 1.02 (0.64-1.61) | P=0.941 |
| Main bronchus | 1.16 (0.83-1.63) | P=0.389 | 1.30 (0.85-1.99) | P=0.220 |
| Overlapping lesion | 0.93 (0.45-1.91) | P=0.842 | 1.63 (0.68-3.91) | P=0.275 |
| Laterality |  |  |  |  |
| Right | Reference |  | Reference |  |
| Left | 0.94 (0.76-1.15) | P=0.547 | 0.94 (0.76-1.16) | P=0.575 |
| Size |  |  |  |  |
| Mean ± SD | 1.01 (1.00-1.02) | P=0.157 | 1.01 (1.00-1.02) | P=0.095 |
| TreatmentType |  |  |  |  |
| Surgery Alone | Reference |  | Reference |  |
| None | 9.98 (5.21-19.1) | P<0.001 | 12.03 (7.80-18.01) | P<0.001 |
| Radiation Therapy Alone | 6.82 (4.14-11.2) | P<0.001 | 6.56 (4.41-9.75) | P<0.001 |
| Chemotherapy Alone | 2.84 (1.55-5.20) | P<0.001 | 5.75 (3.68-8.98) | P<0.001 |
| Radiation+Chemotherapy | 2.51 (1.80-3.50) | P<0.001 | 3.12 (2.25-4.31) | P<0.001 |
| Initial surgery followed by adjuvant treatment | 0.58 (0.44-0.77) | P<0.001 | 1.39 (1.00-1.92) | P=0.050 |
| Induction therapy followed by surgery | 0.50 (0.29-0.85) | P=0.010 | 1.44 (0.81-2.55) | P=0.217 |
| Marital Status |  |  |  |  |
| Married | Reference |  | Reference |  |
| Unmarried/Others | 1.33 (1.08-1.64) | P=0.008 | 1.20 (0.97-1.49) | P=0.092 |

MBI: Main Bronchus Infiltration, P/ATL: Obstructive Pneumonia/Atelectasis, SD: Standard Deviation, AD: Adenocarcinoma, SQCC: Squamous Cell Carcinoma, LCC: Large Cell Carcinoma.
