# Supplementary material for: Exploration and machine learning model development for T2 NSCLC with bronchus infiltration and obstructive pneumonia/atelectasis
Source: Sci Rep. 2024 Feb 27;14:4793. doi: 10.1038/s41598-024-55507-6 (PMC10899628; doi:10.1038/s41598-024-55507-6)
Supplement: Supplementary file 3 — Supplementary Information 3. [file 41598_2024_55507_MOESM3_ESM.docx]

**Supplementary data 3.** Performance of various models in MBI and P/ATL test sets.

Performance of various models in MBI test sets.

| Model | AUC | CE | Accuracy | Recall | Sensitivity | Specificity |
| --- | --- | --- | --- | --- | --- | --- |
| XGBoost | 0.813993589 | 0.229447853 | 0.770552147 | 0.689189189 | 0.689189189 | 0.801011804 |
| Ranger | 0.798717773 | 0.240490798 | 0.759509202 | 0.684684685 | 0.684684685 | 0.787521079 |
| KNN | 0.780117338 | 0.31372549 | 0.68627451 | 0.740740741 | 0.740740741 | 0.674603175 |
| LR | 0.762886833 | 0.284662577 | 0.715337423 | 0.657657658 | 0.657657658 | 0.73693086 |
| ID3 | 0.761143521 | 0.256441718 | 0.743558282 | 0.653153153 | 0.653153153 | 0.777403035 |
| SVM | 0.728734637 | 0.317791411 | 0.682208589 | 0.621621622 | 0.621621622 | 0.704890388 |

Performance of various models in P/ATL test sets.

| Model | AUC | CE | Accuracy | Recall | Sensitivity | Specificity |
| --- | --- | --- | --- | --- | --- | --- |
| XGBoost | 0.853066623 | 0.199813259 | 0.800186741 | 0.73015873 | 0.73015873 | 0.815192744 |
| Ranger | 0.834053198 | 0.21101774 | 0.78898226 | 0.682539683 | 0.682539683 | 0.811791383 |
| LR | 0.817157374 | 0.251167134 | 0.748832866 | 0.724867725 | 0.724867725 | 0.753968254 |
| ID3 | 0.808381024 | 0.220354809 | 0.779645191 | 0.687830688 | 0.687830688 | 0.799319728 |
| KNN | 0.780117338 | 0.31372549 | 0.68627451 | 0.740740741 | 0.740740741 | 0.674603175 |
| SVM | 0.7244688 | 0.31372549 | 0.68627451 | 0.650793651 | 0.650793651 | 0.693877551 |
