# Supplementary material for: Exploration and machine learning model development for T2 NSCLC with bronchus infiltration and obstructive pneumonia/atelectasis
Source: Sci Rep. 2024 Feb 27;14:4793. doi: 10.1038/s41598-024-55507-6 (PMC10899628; doi:10.1038/s41598-024-55507-6)
Supplement: Supplementary file 4 — Supplementary Information 4. [file 41598_2024_55507_MOESM4_ESM.docx]

**Supplementary data 4.** Delong test for each model in MBI and P/ATL.

Delong test in MBI models.

| Model1 | Model2 | P_value |
| --- | --- | --- |
| XGBoost | KNN | <0.001 |
| XGBoost | SVM | <0.001 |
| XGBoost | RF | 0.006 |
| XGBoost | ID3 | <0.001 |
| XGBoost | LR | <0.001 |

Delong test in P/ATL models.

| Model1 | Model2 | P_value |
| --- | --- | --- |
| XGBoost | KNN | <0.001 |
| XGBoost | SVM | <0.001 |
| XGBoost | RF | 0.01 |
| XGBoost | ID3 | <0.001 |
| XGBoost | LR | <0.001 |
