# Supplementary material for: Exploration and machine learning model development for T2 NSCLC with bronchus infiltration and obstructive pneumonia/atelectasis
Source: Sci Rep. 2024 Feb 27;14:4793. doi: 10.1038/s41598-024-55507-6 (PMC10899628; doi:10.1038/s41598-024-55507-6)
Supplement: Supplementary file 5 — Supplementary Information 5. [file 41598_2024_55507_MOESM5_ESM.docx]

**Supplementary data 5.** The ideal number of parameters for each model in MBI and P/ATL.

Optimal parameters for each model in MBI.

| Model | Parameters |
| --- | --- |
| KNN | k=13,distance=1 |
| RF | mtry=3,min.node.size=10,num.trees=100 |
| ID3 | cp=0.001,minsplit=20 |
| SVM | cost=10,gamma=0.1 |
| XGBoost | nrounds=23,max_depth=8,eta=0.1,gamma=0,colsample_bytree=0.6,min_child_weight=1,subsample=0.9,alpha=0,lambda=1 |
| LR | / |

Optimal parameters for each model in P/ATL.

| Model | Parameters |
| --- | --- |
| KNN | k=13,distance=1 |
| RF | mtry=1,min.node.size=5,num.trees=63 |
| ID3 | cp=0.001,minsplit=15 |
| SVM | cost=1,gamma=0.3162277 |
| XGBoost | nrounds=29,max_depth=8,eta=0.1,gamma=0,colsample_bytree=0.6,min_child_weight=1,subsample=0.8,alpha=0,lambda=2 |
| LR | / |
